# The communication of a secondary care diagnosis of autoimmune hepatitis to primary care practitioners: a population-based study

**DOI:** 10.1186/1472-6963-13-161

**Published:** 2013-05-01

**Authors:** Fumi Varyani, Timothy Card, Philip Kaye, Guru P Aithal, Joe West

**Affiliations:** 1 Department of Gastroenterology, Queens Medical Centre, Nottingham University Hospital NHS Trust, Derby Road, Nottingham, NG7 2UH, United Kingdom; 2 Department of Epidemiology and Public Health, Clinical Sciences Building, Nottingham City Hospital, Nottingham, NG5 1 PB, United Kingdom; 3 Department of Pathology, Queens Medical Centre, Nottingham University Hospital NHS Trust, Derby Road, Nottingham, NG7 2UH, United Kingdom

## Abstract

**Background:**

Autoimmune Hepatitis is a chronic liver disease which affects young people and can result in liver failure leading to death or transplantation yet there is a lack of information on the incidence and prevalence of this disease and its natural history in the UK. A means of obtaining this information is via the use of clinical databases formed of electronic primary care records. How reliably the diagnosis is coded in such records is however unknown. The aim of this study therefore was to assess the proportion of consultant hepatologist diagnoses of Autoimmune Hepatitis which were accurately recorded in General Practice computerised records.

**Methods:**

Our study population were patients with Autoimmune Hepatitis diagnosed by consultant hepatologists in the Queens Medical Centre, Nottingham University Hospitals (UK) between 2004 and 2009. We wrote to the general practitioners of these patients to obtain the percentage of patients who had a valid READ code specific for Autoimmune Hepatitis.

**Results:**

We examined the electronic records of 51 patients who had biopsy evidence and a possible diagnosis of Autoimmune Hepatitis. Forty two of these patients had a confirmed clinical diagnosis of Autoimmune Hepatitis by a consultant hepatologist: we contacted the General Practitioners of these patients obtaining a response rate of 90.5% (39/42 GPs). 37/39 of these GPs responded with coding information and 89% of these patients (33/37) used Read code J638.00 (Autoimmune Hepatitis) to record a diagnosis.

**Conclusions:**

The diagnosis of Autoimmune Hepatitis made by a Consultant Hepatologist is accurately communicated to and electronically recorded by primary care in the UK. As a large proportion of cases of Autoimmune Hepatitis are recorded in primary care, this minimises the risk of introducing selection bias and therefore selecting cases using these data will be a valid method of conducting population based studies on Autoimmune Hepatitis.

## Background

Autoimmune hepatitis (AIH) is a chronic liver disease with an immune mediated aetiology. The disease is rare with an annual incidence of 1.9/100000 [1] and has a bimodal age distribution with median age at diagnosis approximately 43 years [2]. It is an uncommon but important disease due to the potential for decompensated cirrhosis and the requirement of an orthotropic liver transplant in otherwise young, healthy people. Recent data from a non-transplant centre in the UK has estimated the 10 year survival to be 82% [3].

The clinical features of AIH are complicated and require a strong clinical suspicion with contributory evidence such as a suggestive liver biopsy, auto-antibodies (anti-nuclear antibodies, anti-smooth muscle cell and anti-liver kidney microsomal antibodies) or an elevated Immunoglobulin G (IgG) titre. Liver biopsy should classically show active interface hepatitis with lymphocytic and plasma-cell infiltrates. There have been several scoring criteria formulated for diagnosis which include the original, revised and simplified [4] criteria by the International Autoimmune Hepatitis group. However, the consensus is that clinical diagnosis is most important [1].

As AIH is a relatively uncommon but important disease, it is difficult to obtain large cohorts of patients to study. Previous studies have used multiple hospital data collection methods, and the largest study so far has obtained 473 patients with Autoimmune Hepatitis [2]. However, these methods are not without potential selection bias and considerable expense. The use of population-based databases may provide an alternative means of obtaining a large cohort of AIH patients. The strengths of databases include prospective, systematic collection of data including exposures, confounders and outcome information. However, their limitations include concerns about the validity of a diagnosis of Autoimmune Hepatitis within the database. The UK General Practice Research Database is one such population-based database. It has over 5 million currently registered patients [5] and it is therefore estimated would provide between 1000–2500 unique patients for studies of the epidemiology of Autoimmune Hepatitis. We have therefore aimed to validate the sensitivity of diagnosis of AIH in electronic primary care data in order to assess its usefulness for studying AIH.

## Methods

### Population

The study population were all patients within the catchment area of Queens Medical Centre, Nottingham University Hospitals UK who were diagnosed by a Consultant Hepatologist with Autoimmune Hepatitis (AIH) between 2004 till 2009. We identified these patients from a list of liver biopsies discussed at a weekly liver biopsy meeting held in the Nottingham University Hospitals, Queens Medical Centre, the larger of the two major hospitals providing Hepatology services to Nottingham, UK). In addition there were a small number of biopsies of patients from nearby District General Hospitals which were reviewed at this meeting.

Nottingham University Hospitals has an electronic system for recording clinic letters and discharge information (NOTIS), which is accessible along with their laboratory, histopathology and microbiology results. Where possible, we obtained these electronic records for those patients with biopsy features of autoimmune hepatitis. We reviewed all patients who had a suggestion of autoimmune hepatitis on their biopsies and who were ever given a clinical label of Autoimmune Hepatitis. Those patients who had available hospital records demonstrating an initial diagnosis of AIH (by a Consultant Hepatologist) and through the course of their clinical encounter were shown not to have an alternative diagnosis became our cohort of patients with Autoimmune Hepatitis. As the course of their disease progressed, some of these patients received an alternative diagnosis or an additional diagnosis as part of an overlap syndrome. We maintained a record of these cases also.

### Validation of clinical diagnosis

If full electronic records were available for the patient, we performed a simplified autoimmune hepatitis score. This score was created by the International Autoimmune hepatitis group in 2008 [4]. In brief its composition is as follows: a maximum of two points are allocated for auto-antibodies, one for an ANA or SMA >=1:40; two for an ANA or SMA >>=1:80, a LKM >=1:40 or SLA positive; IgG >1 times the upper limit of normal (ULN) is allocated one point and >1.1 ULN two points; liver histology that is consistent with autoimmune hepatitis receives one point, and one that is typical of autoimmune hepatitis would receive two; finally the absence of viral hepatitis gains two points. An overall score greater than 6 is classified as probable autoimmune hepatitis, whilst a score greater than 7 is definite autoimmune hepatitis (Table 1). In order to be considered histologically typical three of the following features must be present: interface hepatitis, lymphocytic/lymphoplasmocytic infiltrates in portal tracts and extending into the lobule, emperiopolesis (active penetration by one cell into and through a larger cell) and hepatic rosette formation. A chronic hepatitis with lymphocytic infiltration (without all the features considered to be typical) is considered compatible. As our histopathology department does not regularly examine biopsies for evidence of hepatic rosette formation or emperiopolesis (typical features of autoimmune hepatitis by simplified scoring criteria) we chose to score all our biopsies that had the other two criteria as typical of autoimmune hepatitis. A proportion of biopsies were reviewed by Dr P Kaye (Consultant Histopathologist, Queens Medical Centre, Nottingham University Hospitals, UK) where elements of the histological classification were unclear from the original report and a consensus reached with Dr GP Aithal as to the ranking given.

**Table 1:** **The simplified autoimmune hepatitis criteria [**[4]

**Parameter**	**Cutoff**	**Score**
ANA or SMA	>=1:40	1
ANA or SMA	>=1:80	2
or LKM	>=1:40	2
SLA	Positive	2
IgG	> Upper normal limit	1
	>1.1 Upper normal limit	2
Liver Histology	Compatible with AIH	1
	Typical of AIH	2

The score of each separate parameters are added to provide a simplified scoring system for the diagnosis of autoimmune hepatitis. A score of 6 would be classified as probable AIH, and a score of 7 or more would be definite AIH. A maximum of 2 points will be achieved by addition of all auto-antibodies. For the liver histology, interface hepatitis, lymphocytic/lymphoplamocytic plasma cell infiltrates in portal tracts emperiolysis and hepatic rosette formation were regarded as typical for the diagnosis of AIH. In order to be considered typical, three features had to be present, in order to be classified as compatible, chronic hepatitis with lymphocytic infiltration without all the features considered to be typical were adequate [6]. Abbreviations: *ANA* (Anti-nuclear autoantibodies), *SMA* (Smooth muscle autoantibody), *LKM* (Liver Kidney Microsomal autoantibody) and *SLA* (Soluble Liver antigen), IgG (Immunoglobulin G).

### Assessment of diagnoses coded in general practice for the study population

General Practitioners in the UK use Read codes to code diagnoses. AIH is coded specifically by the two Read codes J63B.00 Autoimmune Hepatitis and J614111 Autoimmune Chronic Active Hepatitis. It can be coded non-specifically by numerous other codes.

We sent the General Practitioners (GPs) of all the patients we had defined as having AIH a letter asking them if these patients had a code for autoimmune hepatitis in their system and if so what code had been used. We also asked how this diagnosis had been communicated to the primary care team. A similar methodology has previously been used by our group for diverticular disease [6].

In the process of identifying patients with AIH, we also identified a cohort of patients who initially receive a diagnosis of AIH, but whose final diagnosis was different. These patients were not scored with the simplified autoimmune hepatitis score. However, letters were sent to their general practitioners asking them if they had READ codes for a range of liver diagnoses (including autoimmune hepatitis, chronic hepatitis, primary biliary cirrhosis and primary sclerosing cholangitis) to assess the possibility of mislabelling them as autoimmune hepatitis. We similarly wrote to those GP surgeries who were caring for patients with any overlap syndrome i.e. PBC/PSC, PBC/AIH or PSC/AIH overlap syndromes.

We carried out this work as an audit registered within the NUH NHS Trust (Audit ID 1582).

## Results

### Study population identified

We identified 51 biopsy reports between 2004 and 2009 in which the possibility of AIH was raised.

After assessment a total of 42 patients were classified consistently as having AIH in secondary care. We were able to obtain demographic and immunological information on the majority of these patients. The approximate age at diagnosis was 48 years, 85% of patients with autoimmune hepatitis were female. 53% of patients with Immunoglobulin G (IgG) levels available had an IgG level greater than the upper limit of normal, 40% of patients with autoantibody information available were Smooth muscle autoantibody (SMA) positive and and 61% were Anti-nuclear autoantibody (ANA) positive (Table 2).

**Table 2:** Demographic information

**Age at diagnosis**	**48 (n=39)**
Sex (% Female)	35/41 (85%)
IgG > ULN (%)	19/36 (53%)
SMA Positivity (%)	15/38 (40%)
ANA Positivity (%)	23/38 (61%)

n=42 patients with AIH. Subsequent denominators for the results depend on availability of results for patients. Abbreviations are as outlined in Table 1.

**Figure 1:**
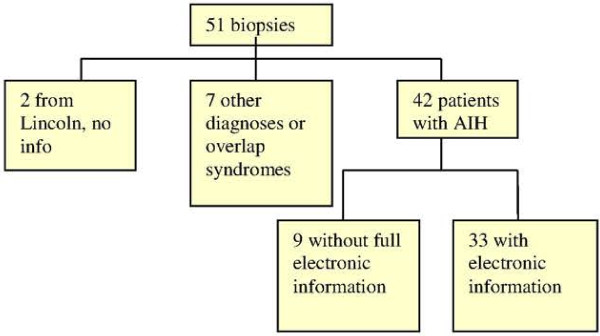
**Flow chart of patients in study and the availability of their electronic records for a simplified AIH score.** We had 51 patients available to study, 2 patients were from another district, and we had no information available about them. 7 patients were subsequently found to have other diagnoses. 42 out of 51 notes examined had a consistent diagnosis of autoimmune hepatitis. Of these, we only had full electronic information available for 79% of these patients.

Of these 42 patients, 33 patients had adequate electronic records available, enabling us to complete a simplified AIH score (Table 1). Of the remaining 9 patients, seven had other diagnoses or an overlap syndrome and two had no electronic notes available because their biopsies came from another region as demonstrated in Figure 1.

### Simplified autoimmune hepatitis score

Using this criteria to categorise biopsies we found 28/33 patients had a score which predicted probable or definite AIH (85%), (Table 3).

**Table 3:** Scores obtained by the simplified autoimmune hepatitis criteria

**Autoimmune hepatitis scoring criteria**	**Number of patients with a particular score**
**>=7 (Definite)**	**16**
**6 (Probable)**	**12**
5	2
4	3
3	0
2	0
1	0

N=33 AIH patients with all available data to calculate a score. We found 28/33 (85%) had a score giving them definite or probable autoimmune hepatitis.

### Overall response rate

We sent 49 letters to General Practitioners and received 46 replies (an overall response rate of 95%). We were unable to send 2 letters because GP address information was not available to us, this was primarily because these biopsies were sent to the liver meeting to provide a second opinion, and then sent back to the local trust. Neither the biopsies nor the demographic information about the patients were retained beyond the meeting for these two biopsies.

### Communication of a diagnosis of AIH

In the study of communication of a secondary care diagnosis of autoimmune hepatitis, we were able to contact the GPs of all 42 patients of our original cohort of autoimmune hepatitis. The overall response rate was 39/42 (93%). 37/39 of the practices responded with coding information, and 33/37 (89%) used Read code 638.00 Autoimmune Hepatitis. None of the practices used the alternative Read code J14111 Autoimmune Chronic Active Hepatitis. Three practices (8%) used non-specific read codes J614100 Chronic Active Hepatitis, and J614.00 for Chronic Hepatitis and one practice used free text to record autoimmune hepatitis (Table 4).

**Table 4:** Responses obtained from the practices

**READ code**	**N**
J638.00 Autoimmune Hepatitis	33/37 (89%)
J614100 Chronic Active Hepatitis	1
J614.00 Chronic Hepatitis	2
Free text box: Autoimmune hepatitis	1

39 practices, out of 42 we wrote to (response rate 93%) and coding method used to document Autoimmune Hepatitis in General Practice. We had 37 responses with coding information available, 2 had left the practice or no information was available.

### Other diagnoses and overlap syndromes

Three patients had an overlap syndrome and four eventually were found to have another diagnosis (Table 5). We received responses from 6/7 of these GPs (86%). 71% of the GP letters available to us were coded accurately. As there are no Read codes for overlap syndromes in General Practice coding systems, GPs would document two separate codes for each of the components of the overlap syndrome (Table 5).

**Table 5:** Patients found to have another diagnosis (or an overlap syndrome)

**Patient**	**Final consultant diagnosis**	**Read code in general practice**
*1*	Ductopenia	J638.00 Autoimmune Hepatitis
*2*	PSC/AIH overlap syndrome	J638.00 Autoimmune Hepatitis
		J661700 Primary Sclerosis Cholangitis
*3*	PBC/PSC overlap syndrome	J616000 Primary Biliary Cirrhosis
		J614.00 Chronic Hepatitis
		Free text in clinic letter ?sclerosing cholangitis
*4*	PBC	J616000 Primary Biliary Cirrhosis
*5*	PBC	J616000 Primary Biliary Cirrhosis
		J638.00 Autoimmune Hepatitis
*6*	PBC/AIH overlap syndrome	No letter received from GP
*7*	PSC	J661700 Primary Sclerosing Cholangitis

## Discussion

More than 300 studies have been performed to assess the positive predictive value of the coding of diseases for the presence of the coded diagnosis. These have in general found high positive predictive values in the order of 90% [7,8]. It is likely that the same will be true for Autoimmune Hepatitis, but that is not what this study has examined. We in contrast have examined the sensitivity of the coded diagnosis of a chronic disease for its presence, and this to our knowledge has very rarely been done before [9].

We assessed the validity of the coding of AIH using a cohort of 42 patients with AIH and 7 patients with other diagnoses or an overlap syndrome whose biopsies were reviewed at the liver biopsy meeting at the Queens Medical Centre, Nottingham, UK, between 2004 and 2009. The diagnosis of AIH is complicated, as essentially there is no single pathognomonic feature [1] and the clinical context is of the utmost importance. Despite there being validated scoring criteria, the consensus of recent British Society of Gastroenterology guidelines on AIH is that this diagnosis requires considerable “clinical expertise” [1] and scoring should be used as an adjunct. We chose to use a consultant diagnosis of autoimmune hepatitis as our gold standard as had Yeoman [10]. We had 42 patients available to us who had a clinical diagnosis of AIH as confirmed by the managing hepatologist, for whom we requested coding information from GPs. We received 39 responses, 37 of the patients had coded or free text information available for the diagnosis of autoimmune hepatitis. Two additional practices wrote back to us to state the patient had left the practice or that they patient was deceased and they no longer held their electronic or paper records (Table 4). Of the 37 GP practices which had information available on the electronic recording of patient data, 89% used a specific READ code for Autoimmune Hepatitis (J638.00 Autoimmune Hepatitis). Others had used non-specific codes which may be interpreted as other forms of liver disease or used free text (Table 4).

We believe therefore that the sensitivity of the coded diagnosis of Autoimmune Hepatitis for the detection of this condition in General Practice is 89% i.e. 89% of all people diagnosed with Autoimmune Hepatitis in hospital get a specific code in electronic primary care records for Autoimmune Hepatitis. This high sensitivity ensures that electronic records are unlikely greatly to underreport the incidence and prevalence of AIH and thus such records are suitable for studying the epidemiology of AIH in databases such as the General Practice Research Database, QResearch or the Health Improvement Network in the United Kingdom. Mediplus is a general practice database used in France, Austria and Germany; Holland uses PHARMO, IPCI and PALGO. In addition there are hospital based databases in England, Spain, Sweden Canada, Australia, Asia (China, Hong Kong, Japan, South Korea, Taiwan and Singapore), Latin America (Brazil, Argentina, Mexico, Venezuela, Peru, Chile). Evidence exists that data from the UKGPRD correlates with other databases in Europe e.g. fracture risk in coeliac disease was found to be increased to the same extent in both the UK GPRD [11] and in the Swedish National Inpatient Register [12] There are also Danish Registries which have been used to look at liver diseases [13].

Seven of the initial fifty patients were subsequently found to have other diagnoses, or overlap syndromes. We wrote to their general practitioners to assess the possibility of a miscoded diagnosis of AIH. If we were considering performing population-based studies, we would be able to exclude most of the overlap and misdiagnosed patients by excluding any person with a code for another form of liver disease as well as autoimmune hepatitis. 71% of these overlap syndromes or those with subsequent diagnoses were accurately coded.

### Limitations

A limitation of this study in the assessment of validity is that it assessed sensitivity of AIH alone. Other measures of diagnostic accuracy or utility include positive predictive value, specificity and negative predictive value. We believe the positive predictive value of AIH to be high in electronic primary care data because this has been shown to be the case in other gastrointestinal diseases such as Coeliac Disease, IBD, Cirrhosis [14] all of which require a similar diagnostic process to AIH including assessment in secondary care and a combination of clinical judgement, laboratory and histological results. Nonetheless further work looking at the positive predictive value of this diagnosis in the GPRD, validating against Hospital Episode Statistics records and/or the clinic letters/discharge information available to the General Practitioners would enhance our confidence in the value of these data. We believe the negative predictive value of a disease such as AIH which has an incidence of approximately 1.9/100 000 would be high simply because it is so uncommon. Other limitations include the lack of data on a therapeutic response, however, as this was a retrospective study there was insufficient information available to come to a valid conclusion about therapy.

All the patients were sourced via the liver biopsy meeting in Nottingham University Hospitals. In Nottingham it is unit policy to biopsy all patients in whom a diagnosis of AIH is made in view of the potentially lifelong therapy which is being embarked upon. Hence in Nottingham only patients who decline a biopsy will not be biopsied. The method chosen to obtain patients for the audit was recommended by the departmental hepatologists as it would be least likely to miss any cases. When compared to a non-transplant centre’s experience of autoimmune hepatitis in Sheffield, only 16 out our 245 cases did not have an initial biopsy, and eventually 7 underwent a biopsy [3].

## Conclusions

Use of electronic primary care data for studying the epidemiology of Autoimmune Hepatitis will be an efficient means of studying this disease, enabling access to large population-based cohorts in order to answer some of the important questions of the contemporary natural history of this disease. Our results suggest, we will through such work be able to precisely quantify the incidence and prevalence of AIH without fear of grossly underestimating the disease as the sensitivity of the recording is so good. Equally, studies of aetiology and consequence should be able to provide much needed insights into this condition. Further work into the positive predictive value of this disease in electronic data is still required.

## Competing interests

We have no competing interests financial or otherwise to declare.

## Authors’ contributions

FV designed the study, collected data, analysed the data and wrote Manuscript. JW Designed the study, supervised and directed the study. TC supervised and directed the study. GA provided the list of patients with liver biopsies and PK helped reanalyse the biopsies for the biopsy criteria for the autoimmune hepatitis criteria. All authors read and approved the final manuscript.

## Pre-publication history

The pre-publication history for this paper can be accessed here:

http://www.biomedcentral.com/1472-6963/13/161/prepub
